# #JACCCardioOnc: Evolution of a Dedicated Social Media Strategy for *JACC: CardioOncology*

**DOI:** 10.1016/j.jaccao.2021.08.004

**Published:** 2021-09-21

**Authors:** Sherry-Ann Brown, Eric H. Yang, Nosheen Reza, Avirup Guha, Roohi Ismail-Khan, Naveen Pemmaraju, Michael G. Fradley, Juan Lopez-Mattei

**Affiliations:** a Cardio-Oncology Program, Division of Cardiovascular Medicine, Medical College of Wisconsin, Milwaukee, Wisconsin, USA; b UCLA Cardio-Oncology Program, Division of Cardiology, Department of Medicine, University of California at Los Angeles, Los Angeles, California, USA; c Division of Cardiovascular Medicine, Department of Medicine, Perelman School of Medicine at the University of Pennsylvania, Philadelphia, Pennsylvania, USA; d Harrington Heart and Vascular Institute, Case Western Reserve University, Cleveland, Ohio, USA; e Cardio-Oncology Program, H. Lee Moffitt Cancer Center and University of South Florida, Tampa, Florida, USA; f Department of Leukemia, Division of Cancer Medicine, The University of Texas MD Anderson Cancer Center, Houston, Texas, USA; g CardioOncology Center of Excellence, Division of Cardiovascular Medicine, Department of Medicine, Perelman School of Medicine at the University of Pennsylvania, Philadelphia, Pennsylvania, USA; h Departments of Cardiology and Thoracic Imaging, University of Texas MD Anderson Cancer Center, Houston, Texas, USA

**Keywords:** cancer, cardio-oncology, social media

Increasing awareness and knowledge surrounding the prevention, screening, diagnosis, and management of cardiovascular injury associated with cancer therapies has ushered in the dedicated subspecialty of cardio-oncology. A rise in cancer survivorship has resulted in growing numbers of aging survivors with cardiovascular disease who also need specific and informed cardiovascular care (1). These phenomena have led to the development of multidisciplinary cardio-oncology programs consisting of cardiologists, oncologists, hematologists, pharmacists, nurses, and other allied health professionals (2). *JACC: CardioOncology* launched in September 2019, and has emerged as the premier journal and essential resource in the field, receiving an impact factor of 6.25 in under 2 years of publication.

In parallel with *JACC: CardioOncology*'s launch, a focused and multimodal social media (SoMe) strategy was created with the overarching goal to augment the dissemination of cardio-oncology knowledge for both patients and health care professionals (1). Both clinicians and patients are increasingly attracted to SoMe sites for up-to-date information on cardio-oncology–related literature (3, 4, 5). Real-time peer review discourse regarding literature or clinical trials is now possible, and patients can find information on possible side effects and treatments (4). Several recent pandemic-related cardio-oncology publications were disseminated on SoMe; within hours, they were being vigorously assessed by the online community (4). Filtering the opinions of experts and non-experts can be difficult with such information overload. With the distinction of being a SoMe influencer in its young existence, *JACC: CardioOncology* has emerged as a leader in reaching patients and professionals alike and directing engaged followers to reliable focused information. Here, we explore the dissemination of *JACC: CardioOncology* publications via our journal SoMe accounts on Facebook, Twitter, and Instagram.

Several studies have analyzed Twitter's potential to contribute toward cardio-oncology's global network (3, 4, 5). The microblogging site has proven helpful for awareness, networking, collaboration, and education through features such as its hashtag campaigns and “Twitter Chat” format (3, 4, 5). Momentum was built on SoMe before the journal’s launch in September 2019; interestingly, the first tweet mentioning #JACCCardioOnc appeared on Twitter on November 24, 2018. As of October 2019, comprehensive journal analytics using the hashtag #JACCCardioOnc via @JACCJournals were available. Our analysis therefore encompasses the period of October 2019 to September 2020 to capture 1 year of journal presence on SoMe. We place these findings in the context of SoMe for all American College of Cardiology (ACC) journals.

During this period, the abundance of media impressions (number of times individuals viewed SoMe posts), clicks, link clicks to JACC.org, saves, comments, quote tweets, retweets, reshares, and likes indicated favorable engagement, particularly regarding cardio-oncology during the pandemic (Figure 1). Although it is challenging to prove that SoMe impressions directly affect cardio-oncology clinical practice, there is much evidence in the nonmedical world of the influence of a posting with a high number of impressions. In cardio-oncology, most respondents to a recent Twitter poll using hashtags #CardioOnc, #CardioOncology, #CardioTwitter reported changing their clinical practice after engaging with journal paper SoMe posts, suggesting that higher rates of impressions may suggest the potential for changing practice.

**Figure 1:**
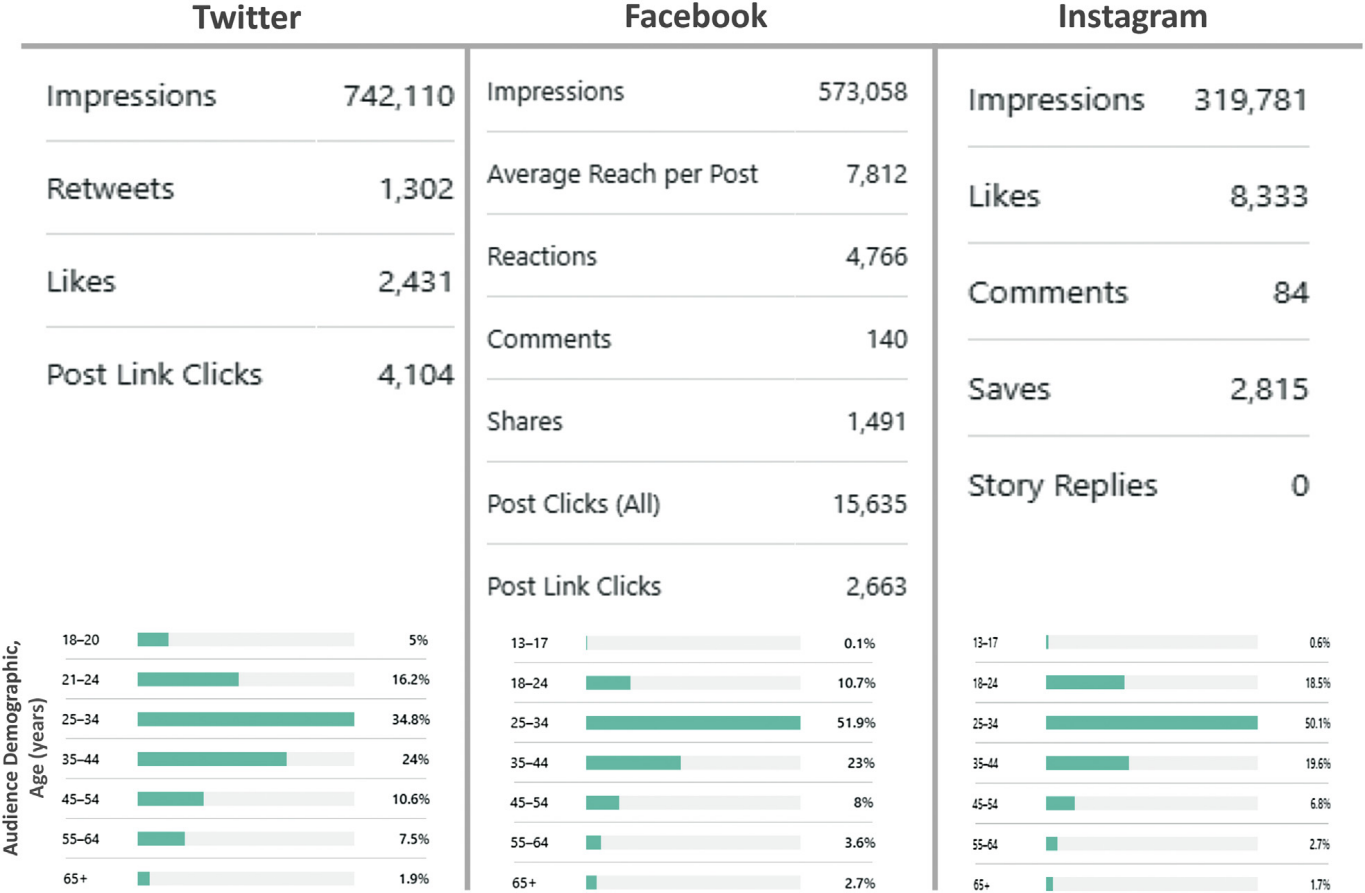
Journal Social Media Engagement Statistics The journal social media posts received high engagement on Facebook, Twitter, and Instagram.

To place this SoMe engagement in context, we have promoted new issues and podcasts on SoMe each quarter. Twitter threads are frequently used, complementing 3-5 posts daily. Twitter’s microblogging interface allows for short communications, is the platform widely used by health professionals in cardio-oncology, and has emerged as an important medical education tool in the Americas and Europe. Disease process diagrams are routinely used on Instagram, with 1 post daily. Instagram is well-suited for cardiovascular image-centric communications. New clinical studies are also shared on Facebook, with 1-3 posts daily. Facebook has the largest global user base; allows for text-based interactions with photo, video, and link embedding capability; and has many engaged followers in Asia. Additionally, and primarily to further engage our international audience, we have leveraged a “live” video journal club series, and cross-promotion of *JACC: CardioOncology* on WeChat with Chinese language translations. Content is also featured on LinkedIn**.**

In 2019, the overall journal Altmetric Attention Score was 1,350, with 2,029 Twitter mentions and 41 Facebook Mentions. The most talked-about paper was “Pre-Diagnosis Exercise and Cardiovascular Events in Primary Breast Cancer: Women's Health Initiative,” with an Altmetric score of 145 (6). In 2020, the overall journal Altmetric Attention Score was 1,835, with 2,722 Twitter mentions and 50 Facebook mentions. The most talked-about paper was “The Novel Coronavirus Disease (COVID-19) Threat for Patients with Cardiovascular Disease and Cancer,” with an Altmetrics score of 152 (7).

To place these data in context, we looked at SoMe analytics for all ACC journals for 2020. As of December, the @JACCJournals Twitter account had >925,000 tweet engagements in 2020 compared with >490,000 in 2019, showing an increase of ∼90%. The total number of times Twitter users saw content was >18.5 million. A review of audience demographics revealed that it was 70% men and 30% women.

As of December 2020, the overall *JACC* Journals Facebook page had ∼120,000 followers, a >9% increase over the previous year. There were >770,000 engagements in 2020 vs >840,000 in 2019, indicating a decrease of 8%, with ∼16.5 million impressions. Demographic review of the *JACC* Journals Facebook page revealed 62% men and 38% women. The top 5 countries in the audience were India, the United States, Mexico, Brazil, and Egypt. The top 5 cities were Mexico City (Mexico), Cairo (Egypt), Bangkok (Thailand), Lima (Peru), and Delhi (India).

The *JACC* Journals’ presence on Instagram was launched in October of 2019. As of December 2020, the page had ∼70,000 followers, with ∼60,000 new followers in 2020. There were >230,000 engagements in 2020, and the total number of times content was seen by users was >8 million. Demographic review revealed 54% men and 45% women; 1% was unspecified. The top 5 countries in the audience were United States, Brazil, India, Mexico, and Colombia. The top 5 cities were Mexico City (Mexico), Sao Paulo (Brazil), New York (United States), Bogota (Colombia), and Buenos Aires (Argentina).

Interestingly, most followers on Twitter, Facebook, and Instagram were men between the ages of 25 and 34 years, although a much larger percentage of women are followers on Instagram compared with Twitter and Facebook. Globally, female physicians remain substantially under-represented in both cardiology (<20%) (8) and oncology (∼30%) (9), which contributes to the sex-based skew in our demographic data on cardio-oncology SoMe engagement. Therefore, looking toward the future, we must build a stronger pipeline of women and other minority groups in cardiology and oncology to funnel more women and other under-represented minority groups into cardio-oncology, thereby increasing the percentage of female cardio-oncologists and other health care professionals with whom we can engage on SoMe. A recent publication indicated that the hashtag created by ACC for women in cardiology (#ACCWIC) is the most frequently used hashtag on Twitter specifically relevant to women in cardiology (10). In fact, the use of several relevant hashtags by female physicians, especially cardiologists, has grown exponentially over recent years for professional development, community building, and advocacy (10). Perhaps our journal can increase the use of these hashtags to expand our reach to female health care professionals in cardio-oncology. In addition to advocacy for women in cardio-oncology, we also want to ensure engagement among both cardiologists and oncologists for our interdisciplinary journal content. We hope for similar trends to those noted for the broader range of hashtags associated with cardio-oncology SoMe posts (eg, #CardioOnc). In 2014-2018, cardiology and oncology conference-related cardio-oncology hashtag tweets increased substantially, with the majority of tweets posted by cardiologists (70%) and oncologists (78%) (11).

Taken together, these metrics indicate that our SoMe engagement continues to grow as we consistently share educational content and expand our presence on various platforms. Notably, several papers suggest an association between journal paper SoMe posts and subsequent journal paper downloads and citations, which drives journal impact factor (12). In January to June 2021, the top Twitter/Facebook posts featured 2 papers that were the top journal papers viewed in the same time period. Such trends have likely contributed to our outstanding impact factor achieved in <2 years.

Our SoMe team aims to increase our international audience, including in the Middle East, Latin America, and East Asia, by 10%. We also intend to increase @JACCJournals engagement/mentions on all of our SoMe platforms. We will continue to employ relevant hashtags and relevant tagging of individuals to increase mentions and engagements, especially among key demographics, including women, early-career cardiovascular professionals, specific clinical areas, and/or key countries/regions. These goals are aligned with *JACC: CardioOncology*, with a vision of becoming the primary scientific source of cardio-oncology globally through publishing high-quality and impactful science. *JACC: CardioOncology* also seeks to be the “go to resource” through the dissemination of knowledge and learning through How to Series, International Perspectives and Leadership Pages, Case Challenges, Viewpoints, State-of-the-Art Reviews, and Primers, along with hosting online international multilingual collaborative journal clubs, podcasts, and webinars.

To maintain momentum, we continue to direct efforts to establish the journal as a premier scientific resource in cardio-oncology. We aim to publish excellent, rigorous, and impactful science to advance clinical medicine and improve patient care. Since the journal’s inception, SoMe has served to augment our reach, putting the new journal on par with other major cardiology journals that have been in existence for a longer time. Our goal is to enhance the global community in cardio-oncology and to continue to use SoMe as a tool to increase our reach.

## Funding Support and Author Disclosures

The authors have reported that they have no relationships relevant to the contents of this paper to disclose.
